# Effect of Mucin and Bicarbonate Ion on Corrosion Behavior of AZ31 Magnesium Alloy for Airway Stents

**DOI:** 10.3390/ma7085866

**Published:** 2014-08-15

**Authors:** Yongseok Jang, Daniel Owuor, Jenora T. Waterman, Leon White, Boyce Collins, Jagannathan Sankar, Thomas W. Gilbert, Yeoheung Yun

**Affiliations:** 1 Engineering Research Center for Revolutionizing Metallic Biomaterials (ERC-RMB), North Carolina Agricultural and Technical State University, 1601 E. Market St, IRC RM 119, Greensboro, NC 27411, USA; E-Mails: pooh3180@hotmail.com (Y.J.); drowuor@gmail.com (D.O.); lgwhite@ncat.edu (L.W.); becollin@ncat.edu (B.C.); sankar@ncat.edu (J.S.); 2 Department of Animal Sciences, North Carolina Agricultural and Technical State University, 1601 E. Market St, Greensboro, NC 27411, USA; E-Mail: jdwaterm@ncat.edu; 3 ACell Inc., 6640 Eli Whitney Drive, Suite 200, Columbia, MD 21046, USA; E-Mail: thomasgilbert@acell.com

**Keywords:** biodegradable metal, magnesium, corrosion, tracheal stent, Gamble’s solution, mucin, bicarbonate ion

## Abstract

The biodegradable ability of magnesium alloys is an attractive feature for tracheal stents since they can be absorbed by the body through gradual degradation after healing of the airway structure, which can reduce the risk of inflammation caused by long-term implantation and prevent the repetitive surgery for removal of existing stent. In this study, the effects of bicarbonate ion (HCO_3_^−^) and mucin in Gamble’s solution on the corrosion behavior of AZ31 magnesium alloy were investigated, using immersion and electrochemical tests to systematically identify the biodegradation kinetics of magnesium alloy under *in vitro* environment, mimicking the epithelial mucus surfaces in a trachea for development of biodegradable airway stents. Analysis of corrosion products after immersion test was performed using scanning electron microscopy (SEM), energy dispersive X-ray spectroscopy (EDX) and X-ray diffraction (XRD). Electrochemical impedance spectroscopy (EIS) was used to identify the effects of bicarbonate ions and mucin on the corrosion behavior of AZ31 magnesium alloys with the temporal change of corrosion resistance. The results show that the increase of the bicarbonate ions in Gamble’s solution accelerates the dissolution of AZ31 magnesium alloy, while the addition of mucin retards the corrosion. The experimental data in this work is intended to be used as foundational knowledge to predict the corrosion behavior of AZ31 magnesium alloy in the airway environment while providing degradation information for future *in vivo* studies.

## 1. Introduction

Patients with airway collapse often have cough, inability to clear secretions, and even respiratory failure requiring mechanical ventilation [1,2] and can lead to dyspnea, hemorrhage and obstructive pneumonia [3]. Airway stents are placed in the airway of patients to restore airway patency caused by stenosis [4,5]. Currently this is accomplished by using permanent stents made from various metals such as stainless steel, cobalt-based super alloy, nitinol (Ni-Ti alloy) or silicone [5,6], however the use of these permanent materials can increase the risk of local inflammation occurring after long-term implantation, which may require open surgery because the stent covered by epithelium are difficult to be removed by conventional bronchoscopic procedure [6,7]. Also, silicon stents can cause obstruction by disturbing the physiological mucociliary function of tracheobronchial epithelium and migration [6]. These complications could be mitigated by removal of the stent [8]. However, a secondary surgery for removal of existing stent results in increased medical expenses and risks.

Biodegradable materials are being actively investigated for airway stents since the need for an airway opening stent is diminished with healing of the airway structure. Especially, biodegradable airway stent can be more useful for pediatric applications. For children, it may be necessary to exchange existing stents for larger ones as they grow. Furthermore, stenting is just needed for 1 to 2 years since their cartilage deficiency can be improved spontaneously [8].

Magnesium alloys have been identified as the promising candidates for biodegradable medical device due to their superior biocompatibility [7,9,10,11]. One of the most desirable characteristics of magnesium alloys as a biodegradable material is its ability to degrade after tissue healing. For this reason, numerous studies have been conducted to understand the biodegradation of magnesium alloys via *in vivo* and *in vitro* tests in various physiological solutions [8,12,13,14]. However, there are only a few studies that focus on the corrosion behavior of magnesium alloys for airway stent applications [8]. It is necessary to systematically identify the corrosion behaviors of magnesium alloys in simulated respiratory fluids for successful development of biodegradable airway stents.

Airway surface liquid (ASL) that covers the surface of airways is composed of a mucus component, a periciliary liquid layer, antimicrobial factors and migratory cells [11,15]. Mucus is composed of approximately 95% water, inorganic and organic ions, lung secretions and proteins [10] and the major macromolecular constituents of mucus are mucins with 80% to 90% carbohydrate by mass [16]. The existence of various ions and protein in mucus can affect the corrosion rate and formation of corrosion products of magnesium alloy. Thus, it is important to confirm the effect of mucin on the corrosion of magnesium alloys within the complex environment of the trachea for development of biodegradable airway stent.

This paper presents the corrosion behaviors of AZ31 magnesium alloy with the concentrations of bicarbonate ion (HCO_3_^−^) and mucin in Gamble solution that mimics the airway surface lining fluid. The fundamental knowledge gained from *in vitro* corrosion studies will provide a basis to help understand the corrosion mechanisms of magnesium alloys under *in vivo* airway environment.

## 2. Results and Discussion

**Figure 1:**
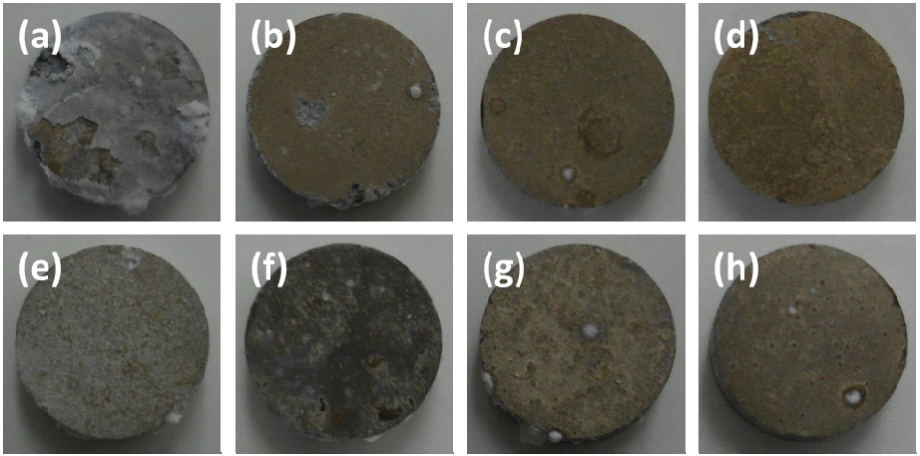
Optical images of corroded surface after immersion test for 10 days in modified Gamble’s solutions: (**a**) Solution #1; (**b**) Solution #2; (**c**) Solution #3; (**d**) Solution #4; (**e**) Solution #5; (**f**) Solution #6; (**g**) Solution #7; (**h**) Solution #8.

Figure 1 shows optical images of the corroded surface after immersion test in modified Gamble’s solutions with different concentrations of NaHCO_3_ and mucin. The surface of AZ31 magnesium alloy after immersion in solution #1 without NaHCO_3_ and mucin was covered with white corrosion products. There was also evidence of delamination of corrosion products from alloy, which was occurred during drying process after immersion test due to their brittle property. After immersion in solution #2, the surface color changed to brown with the presence of localized corrosion at the edges and center parts of the sample. With an increase in concentration of NaHCO_3_, a darker brown color was observed, but there was no evidence of further localized corrosion. The surface color changed to mixture of white and brown and localized corrosion greatly diminished with the addition of 0.1 g·L^−1^ of mucin in solution #1. The surface color changed to a brown increasing the concentration of NaHCO_3_ in solutions. The concentrations of physiological ions in solution can affect the formation of corrosion products and corrosion type such as uninform corrosion and pitting corrosion on the surface of magnesium alloys [17,18].

**Figure 2:**
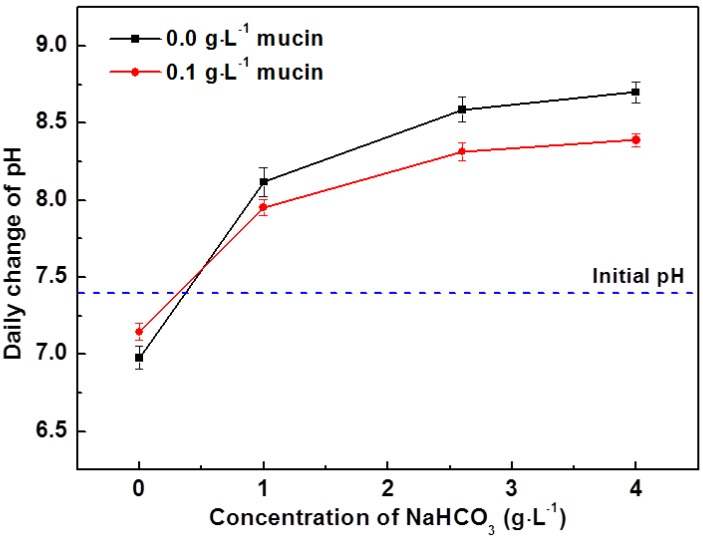
Daily pH change of modified Gamble’s solutions with different concentration of NaHCO_3_ and mucin during immersion of AZ31 magnesium alloys (*n* = 3).

The average daily pH change for solutions during immersion tests of AZ31 magnesium alloy in each solution for 10 days is shown in Figure 2. During immersion tests, the pH of physiological solutions in the presence of magnesium alloys usually increases due to the generation of (OH)^−^ as magnesium alloys corrode [19]. However, the pH of solutions in which NaHCO_3_ was not added decreased during immersion tests even with the addition of mucin. The pH of solution #1 without NaHCO_3_ and mucin decreased to 6.9, while the pH of solution #5 which included 0.1 g·L^−1^ of mucin and no NaHCO_3_ addition, decreased to 7.1. The decrease of solution pH during immersion test of magnesium alloy can be attributed to relative amount of OH^−^ and H^+^ generated by corrosion of alloy and by chemical reactions involved in the formation and transformation of calcium phosphates respectively [17]. In solution #1, the deposition of calcium phosphate more actively occurred on the surface of alloy due to localized pH increase near surface of alloy by weaker buffering effect than other solutions, but the corrosion of alloy can be retarded by the formation of thick calcium phosphates. The addition of NaHCO_3_ in the solution resulted in a substantial increase in pH. The pH of solution #4 increased to 8.7. This indicated that the amount of OH^−^ ions generated from the corrosion of magnesium alloy by carbonate ions was significantly higher than the amount of H^+^ ions generated from the formation and transformation of calcium phosphates in solution [17]. The change of pH increased less with the addition of 0.1 g·L^−1^ mucin in solutions in comparison with solutions that have the same concentration of NaHCO_3_. It is proposed that adsorption of mucin on the surface can retard both the corrosion of magnesium alloys and the rate of hydroxyapatite formation. This explains the suppression of pH change with the addition of mucin. The effects of protein adsorption for the biodegradation of magnesium alloys has been reported by previous studies [14,15,19,20], which can be referred for this study even though most studies used albumin in their experiments. They show that the adsorption of protein can affect the degradation of magnesium alloys, which is dependent with the kind of alloys, the concentration of protein and experimental time. For example, Gu *et al.* [15] confirmed that the corrosion rates of AZ31 and AZ91 decrease with the addition of fetal bovine serum (FBS) in Dulbecco’s Modified Eagle’s Medium (DMEM) after immersion for 7 days, but Mg-Ca alloy shows increased corrosion rate in contrast to AZ alloys. They explained that it is because more stable and compact Al_2_O_3_ layer formed on AZ alloys make protein adhere easier than MgO/Mg(OH)_2_, which can retard the anodic reaction [20], in other hand, increased corrosion rates of Mg-Ca alloys by the addition of FBS in physiological solutions can be explained by chelating effect [7,14,20].

**Figure 3:**
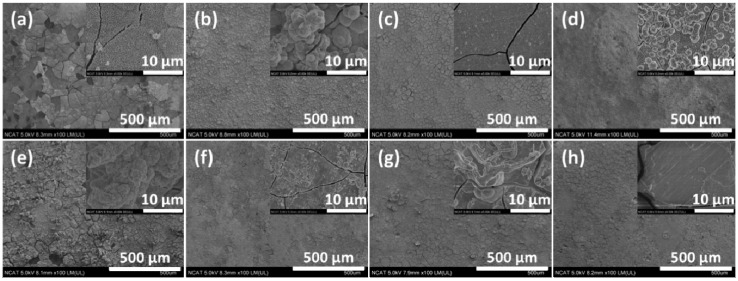
SEM (scanning electron microscope) images of corroded surface of AZ31 magnesium alloys after immersion test for 10 days in modified Gamble’s solutions: (**a**) Solution #1; (**b**) Solution #2; (**c**) Solution #3; (**d**) Solution #4; (**e**) Solution #5; (**f**) Solution #6; (**g**) Solution #7; (**h**) Solution #8.

Figure 3 shows SEM (scanning electron microscope) images on the surface of the samples after immersion tests for 10 days in the modified Gamble solutions. In solution #1, porous corrosion products composed of tiny rod-shaped microstructures formed on the surface, and some corrosion products were chipped off from matrix after immersion test. Corrosion products formed on the surface after immersion test in solution #2 were composed of many globular microstructures, denser than the products observed after immersion in solution #1. The globular microstructures disappeared and increasingly denser corrosion products were formed as the NaHCO_3_ concentration increased (solutions #3 and #4). This result shows that addition of HCO_3_^−^ in solution makes corrosion products dense and improves their adhesive properties on magnesium alloys. The corrosion products formed after immersion in solution #5 were changed to smaller rod-shaped microstructures. Also corrosion product detachment from the matrix in this solution was less than the corrosion products formed after immersion in solution #1. The corrosion products formed on the surface after immersion in solution #8 with the addition of both NaHCO_3_ and mucin were the densest of all tested solutions. Similar precipitation of smaller calcium phosphate was observed by addition of bovine serum albumin (BSA) in 4 times revised simulated body fluid (r-SBF) using a constant-composition double-diffusion device in previous research [21]. It was determined that protein like BSA was co-precipitated with calcium phosphate and had a strong negative effect on the precipitation of calcium phosphates. Studies of adsorptivity of mucin on titanium powders have shown that hydroxyapatite has superior adsorptive strength in comparison to titanium powder alone [22,23]. It was shown that the amount of mucin adsorbed on hydroxyapatite powder and calcium treated titanium powder was about 1.8 times and 3 times higher, respectively, than on titanium powders. Thus the existence of calcium phosphate compounds in corrosion products can accelerate the absorption of mucin on the surface.

Table 1 shows the chemical composition of corrosion products formed on the surface after immersion tests for 10 days in modified Gamble’s solutions with different concentrations of NaHCO_3_ as detected by EDX (energy dispersive X-ray) analysis. The EDX table shows corrosion products formed on the surface of AZ31 magnesium alloy after immersion in solution #1 are mainly composed of Ca, P and O, with a Ca/P ratio of about 1.7 while magnesium was hardly detected in the corrosion products. The Ca/P ratio and morphology of this corrosion product is similar to hydroxyapatite (Ca/P = 1.67) [24,25]. Also, little magnesium in corrosion products was detected in contrast with corrosion products formed in other solutions. It could be attributed to the stable hydroxyapatite with porous structure, which made magnesium ions release in solution more easily. Also, stable hydroxyapatite crystal formed from early stage of immersion might hinder the formation of other corrosion products such as magnesium apatite or magnesium phosphate. However, with increasing the concentrations of NaHCO_3_, the content of Mg increased greatly, but the content of Ca and P decreased. The Ca/P ratios also decreased as they ranged from 0.8 to 1.25 as the concentrations of NaHCO_3_ increased. It is possible that this can be attributed to the presence of magnesium apatite ((Ca_0.86_,Mg_0.14_)_10_(PO_4_)_6_(OH)_2_) [26] or calcium magnesium phosphate (Ca_3_Mg_3_(PO_4_)_4_) [27]. This could also be due to the coexistence of Mg(OH)_2_ with calcium phosphate compounds of low Ca/P ratios like calcium-deficient hydroxyapatite (Ca/P = 1.5–1.67), octacalcium phosphate (Ca/P = 1.33) and amorphous calcium phosphate (Ca/P = 1.2–2.2) [24,28]. 

**Table 1:** Chemical composition of corrosion products formed on the surface after immersion test in solutions for 10 days detected by EDX (energy dispersive X-ray) analysis. (Unit: Atomic %)

Solutions	C	O	Mg	Al	P	Cl	Ca	Zn
#1	6.51	42.10	0.95	0.23	18.42	0.00	31.06	0.25
#2	6.46	46.24	18.04	0.16	12.81	0.06	16.03	0.00
#3	3.89	50.24	21.40	0.34	11.75	0.09	12.16	0.00
#4	5.58	44.94	29.60	0.98	9.96	0.50	8.42	0.00

**Figure 4:**
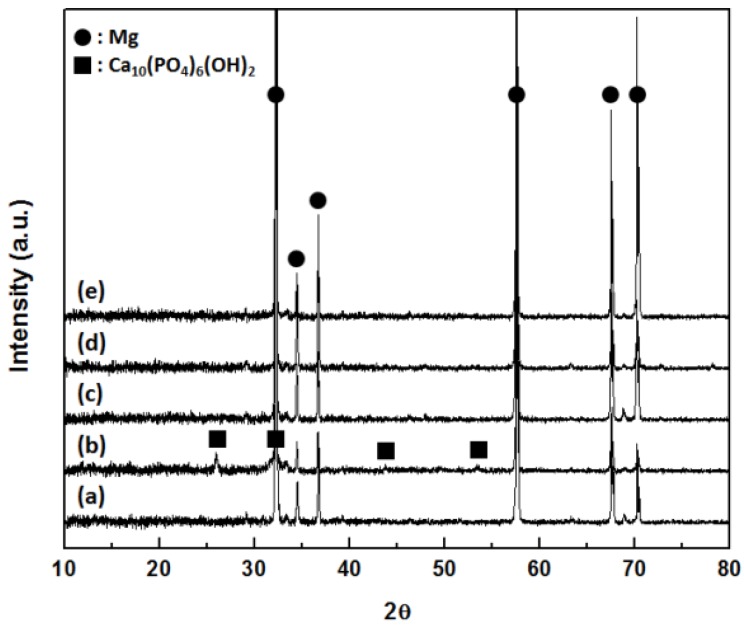
XRD (X-ray diffraction) patterns of AZ31 magnesium alloys after immersion for 10 days in modified Gamble’s solutions with different concentrations of NaHCO_3_: (**a**) AZ31 magnesium alloy; (**b**) Solution #1; (**c**) Solution #2; (**d**) Solution #3; (**e**) Solution #4.

The presence of hydroxyapatite in corrosion products formed on the surface of AZ31 magnesium alloy after immersion was confirmed by XRD (X-ray diffraction) analysis. However, this hydroxyapatite peak was only identified on the surfaces after immersion in solutions #1, and was not observed in corrosion products formed after immersion in others solutions as shown Figure 4. This shows that the existence of HCO_3_^−^ in physiological solution inhibits apatite crystal growth [29]. Even though considerable amounts of Ca, P and Mg were detected by EDX, there was no peak shown in XRD as these corrosion products are considered to be amorphous. Also the relative intensity of Mg(OH)_2_ and calcium phosphate compounds are very weak in comparison with the intensity of Mg peak [17].

**Figure 5:**
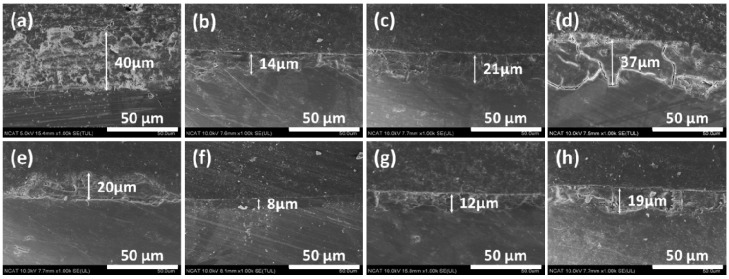
Thickness of corrosion products formed on the surface after immersion for 10 days in modified Gamble’s solutions: (**a**) Solution #1; (**b**) Solution #2; (**c**) Solution #3; (**d**) Solution #4; (**e**) Solution #5; (**f**) Solution #6; (**g**) Solution #7; (**h**) Solution #8.

Figure 5 shows the thickness of corrosion products formed on the surface after immersion in solutions for 10 days. Hydroxyapatite appeared to have precipitated on the surface with a thickness of about 40 μm, but the corrosion products were observed to burrow into the alloy with the addition of NaHCO_3_ and mucin in solutions. The thickness of this corrosion product increased as the concentration of NaHCO_3_ in solution increased. However, the thickness of corrosion product with the addition of 0.1 g·L^−1^ mucin in solution was thinner in comparison with the corrosion products formed in solutions with same concentration of NaHCO_3_ with no added mucin. This implies that the role of HCO_3_^−^ in solution is to accelerate corrosion of magnesium alloy [30] and retard the formation of hydroxyapatite at physiological pH [29]. The formation of calcium phosphate compounds and the corrosion rate of magnesium alloys are sensitive to the concentration of HCO_3_^−^ in physiological solution [17]. The corrosion rate of magnesium usually increases in a solution with concentrations of HCO_3_^−^ above 40 mg·L^−1^ by accelerated dissolution of the magnesium hydroxide (Mg(OH)_2_) protective film [13,30]. Since the concentrations of HCO_3_^−^ in solutions #2 to #4 are over 726 mg·L^−1^, the corrosion of AZ31 magnesium alloy was accelerated by the HCO_3_^−^.

**Figure 6:**
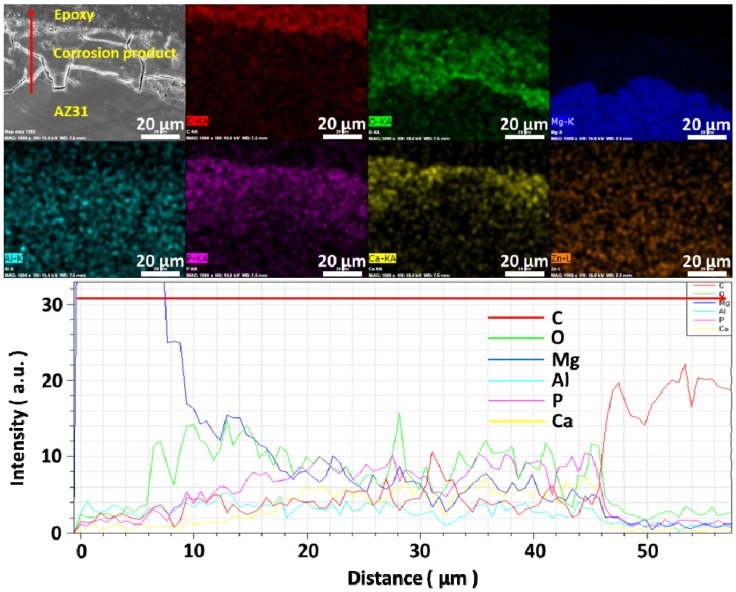
EDX mapping and line analysis of corrosion products formed on the surface after immersion for 10 days in solution #4.

Figure 6 shows the results of EDX mapping and line analysis of corrosion products formed on the surface of AZ31 magnesium alloy after immersion in solution #4 for 10 days. The bottom of corrosion product is mainly composed of Mg and O while the amount of P and Ca gradually increases as the distance from AZ31 substrate increases. It is surmised that the bottom part of the corrosion products are composed of magnesium apatite or calcium magnesium phosphate as well as magnesium hydroxide [26,27]. The top part of the corrosion products is believed to be composed of hydroxyapatite or apatite-like compounds [31] even though it is not detected by XRD.

**Figure 7:**
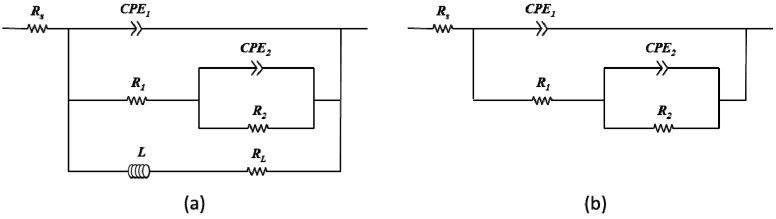
Schematic representation of corresponding equivalent electrical circuits of Electrochemical impedance spectroscopy (EIS) plots acquired in (**a**) Solutions #1 and #5; (**b**) Solutions #2, #3, #4, #6, #7 and #8.

Electrochemical Impedance Spectroscopy (EIS) testing was performed to probe the effect(s) of carbonate ions and mucin on the corrosion behavior of AZ31 magnesium alloys and the resulting data was fit to the equivalent electrical circuits proposed by King *et al.* [32]. In the equivalent electrical circuit of Figure 7, (1) *R*_s_ represents the solution resistance; (2) One of the constant phase element (CPE), CPE_1_ is contributed from the capacitance of double-layer and corrosion product layer; (3) *R*_1_ is represents the pore resistance of corrosion product layer; (4) *R*_2_/CPE_2_ is contributed the adsorption of intermediates induced by charge transfer process; (5) *R*_3_/*L* is contributed from accelerated Mg^2+^ dissolution via fast intermediate steps at actively dissolving areas [20,32,33].

**Figure 8:**
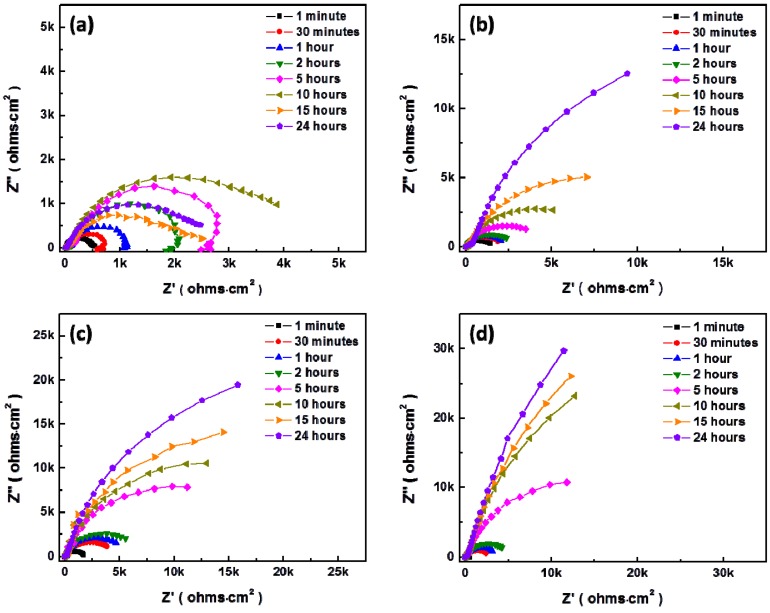
Change of Nyquist plot for AZ31 magnesium alloys in modified Gamble’s solutions with different concentrations of NaHCO_3_ during immersion up to 24 h: (**a**) Solution #1; (**b**) Solution #2; (**c**) Solution #3; (**d**) Solution #4.

**Figure 9:**
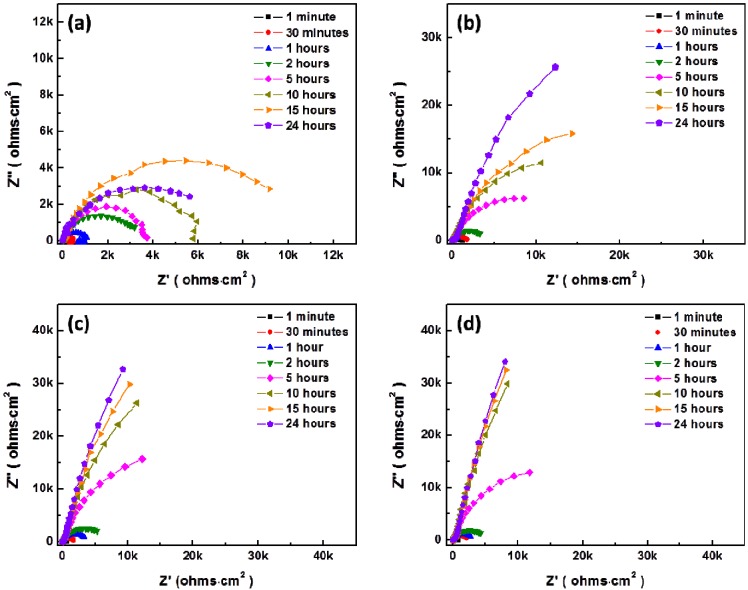
Change of Nyquist plot for AZ31 magnesium alloys with addition of 0.1 g·L^−1^ in modified Gamble’s solutions with different concentrations of NaHCO_3_ during immersion up to 24 h: (**a**) Solution #5; (**b**) Solution #6; (**c**) Solution #7; (**d**) Solution #8.

**Figure 10:**
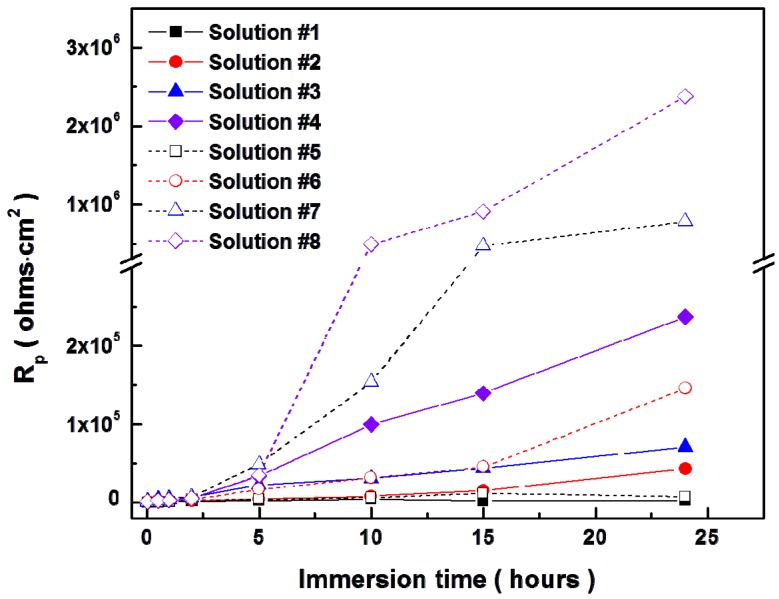
Temporal change of (**a**) coating resistance (*R*_c_) by corrosion products and (**b**) charge transfer resistance (*R*_t_) for AZ31 magnesium alloys during immersion in modified Gamble’s solutions up to 24 h.

Figure 8 and Figure 9 show the temporal changes of Nyquist plots for AZ31 magnesium alloys during immersion in modified Gamble’s solutions with different concentrations of NaHCO_3_ and mucin up to 24 h at 37 °C respectively. Polarization resistance, *R*_p_, of Nyquist plots which include the inductive response was determined by 
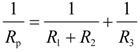
 after fitting with Figure 7a as suggested by King *et al.* [32], and *R*_p_ of Nyquist plots without inductive response was calculated by *R*_p_ = *R*_1_ + *R*_2_ after fitting with Figure 7b, which were summarized in Figure 10. In solution #1 and #5 which did not include NaHCO_3_, the inductive response was observed and *R*_p_ was increased up to 10 and 15 h respectively and then decreased during immersion. However, *R*_p_ increased with increasing the concentration of NaHCO_3_ in solution during immersion, and *R*_p_ more sharply increased with addition of 0.1 g·L^−1^ mucin than in those solutions that did not. Relative lower increment of *R*_p_ in solution #1 and #5 in comparison with other solution and the inductive response could be caused by the formation of porous corrosion products on the surface of AZ31 magnesium alloy. The increment of *R*_p_ and no inductive response with increase of NaHCO_3_ in solution was caused by formation of dense corrosion product layer [32,33]. More increment of *R*_p_ with addition of mucin in solution although the corrosion product layer is thinner as shown in Figure 5 might result from the adsorption of mucin on the surface and the formation of more dense corrosion product layer than the corrosion product layer formed in solution without mucin.

**Figure 11:**
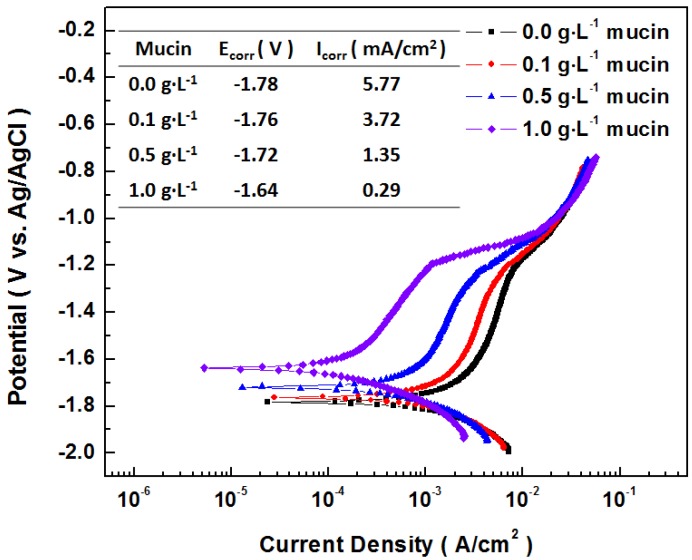
Potentiodynamic polarization curves for AZ31 magnesium alloy in Gamble solution (Solution #3) with different concentrations of mucin.

Figure 11 shows potentiodynamic polarization curves for AZ31 magnesium alloy in Gamble solution (Solution #3) with different concentrations of mucin. The corrosion potentials increased and corrosion current density (*i*_corr_) remarkably reduced with the increase of mucin in the solution. It implied that mucin can effectively decrease the corrosion rate of magnesium by acting as a barrier shield from the initial stages of corrosion. This results in the formation of denser corrosion products. These results can support the reason why *R*_p_ increased by addition of mucin in Figure 9. Although we could not find literature related to the effect of mucin on the corrosion of magnesium alloys, it has been shown that mucin in physiological solutions retarded the corrosion of other metallic biomaterials such as silver alloys through electrochemical analysis [34]. Also, the effect of absorption of other proteins on corrosion resistance has been identified on magnesium alloys [20,35] and other biomaterials [36,37].

**Figure 12:**
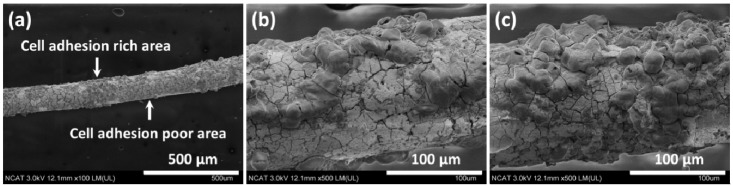
SEM images of Porcine Tracheal Epithelial (PTE) cells adhered on the surfaces of pure magnesium wire after the cell adhesion test for 12 h: (**a**) Low magnification image of pure magnesium wire after cell adhesion test; (**b**) High magnification image of cell adhesion poor area; (**c**) High magnification image of cell adhesion rich area.

Biocompatibility of magnesium as airway stent material was assessed using Porcine Tracheal Epithelial (PTE) cells to determine the cell responses on the surfaces of pure magnesium wire. Figure 12a shows many cells were well attached but distributed non-uniformly on the wire which may be due to the cylindrical morphology of the wire. Cells were attached closely together showing good interaction between cells and corrosion products formed on the wire as shown Figure 12b,c. Cell adhesion can be affected by various factors such as hydrogen gas evolution, increment of localized pH and released ions and corrosion products. Thus, good cell interaction on the surface of magnesium wire means that the increment of localized pH and magnesium ions produced from stent dissolution is not expected to lead to toxic reactions during biodegradation.

## 3. Experimental Section

### 3.1. Sample Preparation

Cylindrical as drawn AZ31 magnesium alloys with diameter of 6.35 mm (Goodfellow Corp., Coraopolis, PA, USA), which have the nominal mass composition of 96% Mg, 3% Al and 1% Zn, were cut with a height of 2 mm. Samples were polished up to 1200 grit using SiC paper and rinsed with isopropanol for the immersion test. The electrochemical samples were embedded in epoxy resin to expose a working area of 0.317 cm^2^ to test solutions. Electrical connection was provided via a copper wire. In order to ensure consistent surface roughness for all samples, the exposed surface was also polished in the same manner as the immersion test samples.

### 3.2. Test Solution

Evaluation of corrosion behavior of the AZ31 magnesium alloy was performed using both electrochemical and immersion testing in modified Gamble’s solutions that mimics the biological environment in the airway [38]. When preparing modified Gamble’s solutions, the chemicals were added in the order presented in Table 2 to avoid salt precipitation. Porcine stomach mucin (Sigma-Aldrich, St. Louis, MO, USA) was later added in the modified Gamble’s solution to create an *in vitro* model of the epithelial mucus surfaces mimicking the epithelial surface along the trachea. Solutions in Table 3 were used for the immersion and electrochemical tests to confirm their effects. The solutions were continuously stirred at room temperature for 30 min until a homogeneous solution was obtained. The pH of each solution was adjusted using 0.1 M HCl and 0.1 M NaOH to obtain a pH of 7.4 ± 0.05.

### 3.3. Immersion Tests

Immersion tests were conducted in an isotemp incubator (Fisher Scientific 1602D, Dubuque, IA, USA) at a general atmospheric environment of 37 °C for 10 days. The solutions were changed every day to minimize the impact of pH for corrosion of AZ31 magnesium alloy and the pH of each solution was measured before changing the solution using a pH meter (Eutech instruments, Oakton^®^ pH2100, Singapore). The ratio of volume of solution to area of sample was 300 mL·cm^−2^.

**Table 2:** Concentrations of chemicals used to prepare Gamble’s solution [38].

Chemicals	Chemical Formula	Concentration
Magnesium Chloride	MgCl_2_	0.095 g·L^−1^
Sodium Chloride	NaCl	6.019 g·L^−1^
Potassium Chloride	KCl	0.298 g·L^−1^
Disodium Hydrogen Phosphate	Na_2_HPO_4_	0.126 g·L^−1^
Sodium Sulfate	Na_2_SO_4_	0.063 g·L^−1^
Calcium Chloride Dihydrate	CaCl_2_·2H_2_O	0.368 g·L^−1^
Sodium Acetate	CH_3_COONa	0.574 g·L^−1^
Sodium Hydrogen Carbonate	NaHCO_3_	2.604 g·L^−1^ *
Sodium Citrate Dihydrate	C_6_H_5_Na_3_O_7_·2H_2_O	0.097 g·L^−1^

* The concentration of Sodium Hydrogen Carbonate was changed as Table 2.

**Table 3:** Modified Gamble’s solutions with different concentrations of NaHCO_3_ and mucin.

	NaHCO_3_ (g·L^−1^)	0	1	2.6	4
Mucin (g·L^−1^)					
0		Solution #1	Solution #2	Solution #3	Solution #4
0.1		Solution #5	Solution #6	Solution #7	Solution #8

### 3.4. Electrochemical Tests

The electrochemical corrosion behavior of the AZ31 magnesium alloy was investigated in the different solutions in Table 2 by electrochemical impendence spectroscopy (EIS) using a Potentiostat/Galvanostat/ZRA (Gamry Instruments, Warminster, PA, USA). A three-electrode cell with the sample as the working electrode, Ag/AgCl electrode as reference electrode and platinum wire as counter electrode was used. The EIS data were recorded from 100 kHz to 500 mHz with a 10 mV sinusoidal perturbing signal. The temporal EIS data was monitored as a function of immersion time and the data was recorded for 24 h. Potentiodynamic polarization curves were obtained by scanning the potential from −0.2 V/E_OCP_ to 1 V/E_OCP_ with a scanning rate of 0.5 mV·s^−1^. Electrochemical tests were carried out at 37 °C which was controlled by a water bath (Fisher Scientific, Marietta, OH, USA).

### 3.5. Corrosion Characterization

The surface morphology and cross-sectional images were observed using SEM (SU8000, Hitachi, Tokyo, Japan). The chemical composition and crystal structure of corrosion products on the surface of the samples were analyzed using XRD (Bruker D8 Discover, Karlsruhe, Germany) and EDX (Bruker AXS5350, Berlin, Germany) installed with SEM after ion beam coating to improve image clarity.

### 3.6. Adhesion Test of Porcine Tracheal Epithelial (PTE) Cells

For cell adhesion experiments, as drawn high purity magnesium wires (99.9%, Goodfellow Corp.) with 125 μm diameter and 1 cm length were affixed to glass discs with polydimethylsiloxane (PDMS) and sterilized by UV light for 15 min prior to cell exposure. Cryopreserved porcine airway epithelial cells (a gift from Dr. Jenora Waterman) were thawed in a 37 °C water bath for 1–2 min and resuspended in 9 mL of porcine complete medium (50:50 DMEM/Ham’s F12 supplemented with 2% fetal bovine serum, 100 U·mL^−1^ penicillin, 0.1 mg·mL^−1^ streptomycin, 5 μg·mL^−1^ amphotericin B, 0.1 mg·mL^−1^ l-glutamine, 5 ng·mL^−1^ epidermal growth factor, 5 μg·mL^−1^ transferrin, 5 μg·mL^−1^ insulin, 0.1 pg·mL^−1^ retinoic acid, and 20 ng·mL^−1^ triiodothyronine). Cell viability was determined using a TC20 Automated Cell Counter (Bio-Rad, Carlsbad, CA, USA) and seeding density was adjusted to 3.0 × 10^5^ viable cells/well in 1.0 mL media prior to seeding in 24-well cell culture cluster plates (Corning Inc., Corning, NY, USA) included magnesium wires. Culture vessels were placed in a 37 °C incubator with humidified air infused with 5% CO_2_ for 12 h. Following incubation, the cell culture media was aspirated and the samples were rinsed three times with 1× phosphate-buffered saline (PBS) to remove non-adherent cells. The cells that adhered to wire were then fixed in 4% paraformaldehyde for 1 h and gradually dehydrated using 50%, 70%, 90% and 100% ethanol for 15minutes each. Analysis was performed by SEM observation after ion beam coating.

## 4. Conclusions

The effects of bicarbonate ions and mucin on the degradation behavior of AZ31 magnesium alloys have been systematically studied in modified Gamble’s solution with different concentrations of carbonate ions and addition of mucin. The bicarbonate ions in Gamble’s solution are shown to not promote magnesium dissolution but form thick and dense corrosion products. Thus, the concentration of bicarbonate ion in Gamble’s solution is suspected to pose great influence on *in vitro* corrosion performance of biomedical magnesium alloys. The addition of porcine stomach mucin into the Gamble’s solution inhibited the corrosion of AZ31 magnesium alloy by adsorption on the surface. Our results help to provide a better understanding of the degradation mechanism of magnesium alloy in bronchial-related physiological environments and should be helpful to clinical applications of biomedical magnesium alloys.
